# Successful perioperative management of living-donor liver transplantation for a patient with severe methylmalonic acidemia: a case report

**DOI:** 10.1186/s40981-018-0221-y

**Published:** 2018-12-27

**Authors:** Akiko Hirotsu, Eriko Kusudo, Natsumi Mori, Yoshimitsu Miyai, Kengo Suzuki, Shuji Kawamoto, Kazuhiko Fukuda

**Affiliations:** 1 Department of Anesthesia, Kyoto University Hospital, 54, Shogoinkawahara-cho, Sakyo-ku, Kyoto, 606-8507 Japan; 2 Department of Anesthesia, Osaka Red Cross Hospital, 5-30, Fudegasaki-cho, Tennoji-ku, Osaka, 543-8555 Japan

**Keywords:** Methylmalonic acidemia, Liver transplantation, Metabolic acidosis, Perioperative management

## Abstract

**Background:**

Methylmalonic acidemia (MMAemia) is a rare hereditary disease affecting organic acid metabolism. It causes recurrent metabolic acidosis and secondary mitochondrial dysfunction, resulting in a poor prognosis. Liver transplantation (LT) has been performed to facilitate the metabolism of organic acids and improve the prognosis of MMAemia. However, there have been few reports on perioperative management of LT.

**Case presentation:**

A 22-month-old female with severe MMAemia was scheduled to receive LT to relieve recurrent metabolic acidosis despite dietary and pharmacological treatment. General anesthesia was maintained without propofol or nitrous oxide, which can worsen MMAemia-induced metabolic acidosis during anesthesia for LT. Strict metabolic and respiratory management enabled the operation to be successfully performed without metabolic acidosis.

**Conclusion:**

Perioperative management of LT for MMAemia is challenging for anesthesiologists because of the possibility of serious metabolic acidosis. We succeeded in preventing metabolic decompensation by avoiding the use of propofol and nitrous oxide.

## Background

**Fig. 1:**
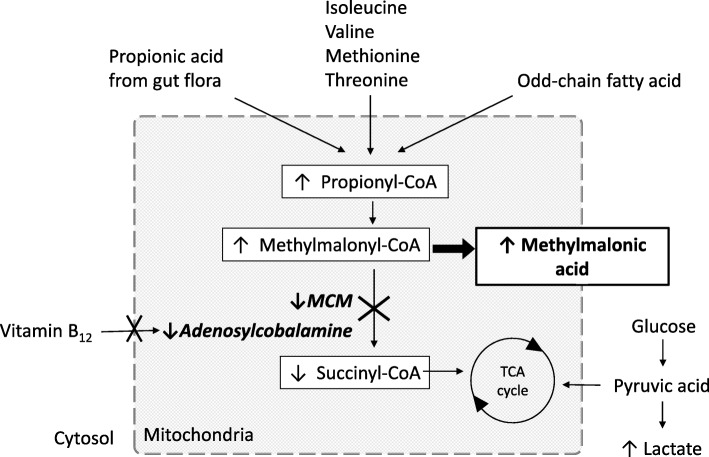
Metabolic pathway of methylmalonic acidemia. Methylmalonic acid (MMA) accumulates systemically because of intracellular methylmalonyl-CoA mutase (MCM) or vitamin B_12_, the coenzyme of MCM

Methylmalonic acidemia (MMAemia) is a rare hereditary disease caused by the accumulation of methylmalonic acid (MMA) due to metabolic disorders related to methylmalonyl-CoA mutase (MCM) or vitamin B_12_, the coenzyme of MCM [1]. MMAemia can affect multiple organ systems, including the liver, kidney, and central nervous system, and the patient often suffers from vomiting, seizures, developmental retardation, and metabolic acidosis. Dietary and pharmacological treatment should be started as soon as the patient is diagnosed with MMAemia because several amino acids, odd-chain fatty acids, and some intestinal florae can facilitate MMA accumulation (Fig. 1). However, the patient often suffers from life-threatening metabolic acidosis requiring emergency treatment characterized by vomiting and elevated ammonia (NH_3_), following hypercatabolism due to febrile illness and inappropriate nutrition [2]. In 50% of severe cases, the patient does not survive beyond 3 years of age because of severe metabolic acidosis and its related symptoms. Recently, liver transplantation (LT) has been performed as supportive therapy for children with severe MMAemia to improve MMA metabolism through the action of the transplanted healthy liver [3]. However, there are few reports on perioperative management of LT for MMAemia [4].

## Case presentation

A 22-month-old female (70 cm, 9.9 kg) with MMAemia was admitted to our hospital to receive living donor LT (LDLT) from her mother. Three days after her normal birth, metabolic acidosis and seizures appeared, and genetic tests revealed a MCM defect, which is one of the most common types of abnormalities in Japanese people. Despite a protein-restricted diet, vitamin B_12_ and l-carnitine supplements, and treatment with anticonvulsant agents, her symptoms were poorly controlled. She was transferred to our hospital with continuous infusion of glucose, vitamin B_12_, and l-carnitine. Her preoperative arterial blood gas (ABG) data were within normal ranges (Table 1). Preoperative plasma MMA (P-MMA) was 157 nmol/ml (normal range 0.35 ± 0.22 nmol/ml). Hepatic and renal functions were within the normal ranges.

**Table 1:** Preoperative and intraoperative arterial blood gas data. Normal range of ammonia (NH_3_) is 20–60 μg/dl

	Pre-operation	At the time of operation	Before reperfusion	2 h after reperfusion	End of operation
pH	7.373	7.377	7.231	7.332	7.344
PaCO_2_ (mmHg)	42	34.4	35.6	29.6	33.3
HCO_3_ (mmol/l)	23.9	19.8	14.6	15.3	17.7
Base excess	− 1.3	− 4.8	− 11.9	− 9.4	− 7.1
Lactate (mg/dl)	23.8	53.2	54.3	30	33
NH_3_ (μg/dl)	55	48	139	58	52
Glucose (mg/dl)	132	173	192	200	237

On the day of the operation, after fasting for 6 h, the patient was transferred to the operating room with continuous glucose, vitamin B_12_, and l-carnitine infusion. General anesthesia was induced and maintained with sevoflurane, fentanyl, remifentanil, and rocuronium. After tracheal intubation, a catheter for continuous hemodiafiltration (CHDF) was inserted to facilitate further intraoperative treatment of metabolic acidosis. Although metabolic acidosis with elevated lactate and NH_3_ occurred during the anhepatic phase, all improved promptly after graft reperfusion (Table 1). LDLT was completed without the use of CHDF or sodium bicarbonate administration for metabolic acidosis. The operation time and anesthetic time were 9 h 45 min and 12 h 40 min, respectively. The total fluid infusion was 1175 ml, including transfusion of 1 unit of red blood cells and 1 unit of fresh frozen plasma. Total urine output was 135 ml, and a small amount of bleeding occurred.

The patient was transferred to the ICU under sedation with midazolam. She was extubated on postoperative day (POD) 3, and P-MMA improved to 18.7 nmol/ml on POD 7. She was discharged on POD 80.

## Discussion

The guidelines for diagnosis and management of MMAemia suggest that LT should be considered in patients with MMAemia who suffer frequent metabolic acidosis despite dietary and medical treatment [1]. A report on 14 cases of LT for MMAemia in a single institute in Japan showed that severe metabolic acidosis occurred in 3 cases at the start of the operation, and death occurred perioperatively in 2 of these 3 cases [4]. Thus, metabolic acidosis should be prevented during LT, even though the prognosis of MMAemia after LT has recently been improved [4].

In a patient with MMAemia, MMA accumulation can cause metabolic acidosis and secondary mitochondrial dysfunction. In contrast to the normal situation, in which the tricarboxylic acid (TCA) cycle is supplied with glucose and succinyl-CoA, in MMAemia succinyl-CoA deficiency causes operation of the TCA cycle to depend mostly on glucose, leading to adenosine triphosphate (ATP) deficiency. Lactate tends to accumulate, followed by lactate acidosis, because the ATP deficiency is compensated by conversion of pyruvate to lactate under anaerobic conditions. NH_3_ can also accumulate because the urea cycle, an ATP consumption pathway in hepatic cells, cannot efficiently process urea. To make matters worse, anaerobic metabolism in the liver graft during the anhepatic phase of LT and the following ischemic graft reperfusion can facilitate metabolic acidosis. In contrast, carnitine plays a beneficial role in patients with MMAemia by binding to propionyl-CoA in liver cells, which facilitates its excretion as propionylcarnitine and prevents accumulation of MMA [5].

We took the pathophysiology of MMAemia into consideration in planning for perioperative management of LT. First, we continued supplementation of glucose, vitamin B_12_, and l-carnitine. Second, we prepared for CHDF to facilitate treatment of metabolic acidosis as needed. Finally, we avoided the use of nitrous oxide (N_2_O) and propofol. N_2_O inhibits adenosyl cobalamin, the cofactor of MCM, through irreversible oxidation of cobalt atoms in vitamin B_12_, and can increase MMA accumulation in patients with MMAemia [6]. Propofol may not harm the patient with MMAemia, considering that the metabolites of propofol and its emulsion are not turning into the precursor of MMA [7], and a previous report had described the effectiveness and safety of the brief use of propofol [8]. However, propofol can inhibit the mitochondrial electron transport system and coupling of oxidative phosphorylation [9] and can exacerbate mitochondrial dysfunction in MMAemia. Furthermore, prolonged and high-dose propofol administration can cause propofol infusion syndrome, characterized by metabolic acidosis, rhabdomyolysis, and liver failure. Although our management could prevent acidosis during LT, further investigation is required for the establishment of safe perioperative management of the patients with MMAemia [9].

The long-term prognosis of MMAemia after LT is unclear because compromised mitochondrial function in renal cells can cause future renal failure [10]. Besides, in LDLT, MCM activity in a graft from a related donor might be lower than that of a non-related donor due to the heterozygous abnormality in the related donor. Thus, long-term observation is needed in these cases.

## Conclusion

Perioperative management of LT for MMAemia is challenging for anesthesiologists because serious metabolic acidosis can occur. We successfully prevented metabolic acidosis in the perioperative period by cautious planning for management of the patient based on the pathophysiology of MMAemia.

## Abbreviations

ABG: Arterial blood gas
ATP: Adenosine triphosphate
CHDF: Continuous hemodiafiltration
LDLT: Living donor LT
LT: Liver transplantation
MCM: Methylmalonyl-CoA mutase
MMA: Methylmalonic acid
MMAemia: Methylmalonic acidemia
N_2_O: Nitrous oxide
NH_3_: Ammonia
P-MMA: Plasma MMA
POD: Postoperative day
TCA: Tricarboxylic acid
